# *Streptococcus pneumoniae* Interacts with pIgR Expressed by the Brain Microvascular Endothelium but Does Not Co-Localize with PAF Receptor

**DOI:** 10.1371/journal.pone.0097914

**Published:** 2014-05-19

**Authors:** Federico Iovino, Grietje Molema, Jetta J. E. Bijlsma

**Affiliations:** 1 Department of Medical Microbiology, University Medical Center Groningen, University of Groningen, Groningen, The Netherlands; 2 Department of Pathology & Medical Biology, Medical Biology section, University Medical Center Groningen, University of Groningen, Groningen, The Netherlands; Biological Research Centre of the Hungarian Academy of Sciences, Hungary

## Abstract

*Streptococcus pneumoniae* is thought to adhere to the blood-brain barrier (BBB) endothelium prior to causing meningitis. The platelet activating factor receptor (PAFR) has been implicated in this adhesion but there is a paucity of data demonstrating direct binding of the bacteria to PAFR. Additionally, studies that inhibit PAFR strongly suggest that alternative receptors for pneumococci are present on the endothelium. Therefore, we studied the roles of PAFR and pIgR, an established epithelial pneumococcal receptor, in pneumococcal adhesion to brain endothelial cells in *vivo*. Mice were intravenously infected with pneumococci and sacrificed at various time points before meningitis onset. Co-localization of bacteria with PAFR and pIgR was investigated using immunofluorescent analysis of the brain tissue. *In vitro* blocking with antibodies and incubation of pneumococci with endothelial cell lysates were used to further probe bacteria-receptor interaction. *In vivo* as well as *in vitro* pneumococci did not co-localize with PAFR. On the other hand the majority of *S. pneumoniae* co-localized with endothelial pIgR and pIgR blocking reduced pneumococcal adhesion to endothelial cells. Pneumococci physically interacted with pIgR in endothelial cell lysates. In conclusion, bacteria did not associate with PAFR, indicating an indirect role of PAFR in pneumococcal adhesion to endothelial cells. In contrast, pIgR on the BBB endothelium may represent a novel pneumococcal adhesion receptor.

## Introduction

*Streptococcus pneumoniae* (the pneumococcus) is the main causative agent of bacterial meningitis in Europe and in the USA [1], [2] and is thought to invade into the brain via the bloodstream by crossing the vasculature of the blood-brain barrier (BBB) [3], [4]. The platelet-activating factor receptor (PAFR) is implicated in pneumococcal adhesion to endothelial cells [5], [6], [7]. *In vitro* blocking and transfection studies and most *in vivo* experiments using PAFR^−/−^ mice clearly indicate that PAFR contributes to the development of invasive pneumococcal disease (IPD) [5], [6], [7], [8]. The question that still remains is whether *S. pneumoniae* binds directly to PAFR. When PAFR is genetically deleted or chemically inhibited, pneumococci still adhere to and invade human cells and cause infections in mice [5], [6], [7] indicating that *S. pneumoniae* can engage alternative receptors [8]. One candidate might be the poly immunoglobulin receptor (pIgR), which is known to bind to pneumococci in human nasopharyngeal epithelial cells [9], [10]. PIgR was previously shown to be expressed in neurons [11], [12], [13], but was not detected in brain endothelial cells [9]. The aim of this study was to investigate the roles of PAFR and pIgR in *S. pneumoniae* adhesion to brain endothelial cells in a bacteremia-derived meningitis model.

Immunofluorescent analysis performed on brain tissue from infected mice, indicates that direct interaction of *S. pneumoniae* with PAFR is unlikely to occur *in vivo*. The same analysis in combination with *in vitro* data demonstrated that pIgR is expressed on brain vascular endothelium and could act as a novel adhesion receptor for *S. pneumoniae* on the BBB.

## Materials and Methods

### Ethics statement

All experiments involving animals were performed in strict accordance with Dutch legislation on animal experiments (“Wet op de dierproeven”, 1977; modified in 1996 with implementation of the European guidelines 86/609/EEG and “Dierproevenbesluit 1985”) with the prior approval of and in accordance with guidelines of the Institutional Animal Care and Use Committee of the University of Groningen (DEC nr. 6152A). Since umbilical cords are usually discarded after birth, anonymous sampling does not need formal ethical committee approval (according the “Code of Good Use” of waste material). Pregnant women are informed during pregnancy that waste-material may be used anonymously for research, and that they can refuse.

### Cell lines, primary cells and culture conditions

Human Brain Microvascular Endothelial Cells (HBMEC) [14] (obtained from Dr. K.S. Kim) were cultivated as previously described [14]. Detroit [15], A549 [16] and Beas2b cells [17] (obtained from Molecular Virology Department, UMCG) were cultivated in accordance to the American Type Culture Collection (ATCC) guidelines. Human Umbilical Vein Endothelial Cells (HUVEC) (obtained from the Endothelial Cell Facility, UMCG) were cultivated as previously described [18].

### Bacterial strains and growth conditions

Encapsulated *S. pneumoniae* TIGR4 [19] was grown in Todd-Hewitt broth (Oxoid Thermo Scientific, Basingstoke, United Kingdom), un-encapsulated TIGR4 was grown in M17 medium (Oxoid Thermo Scientific) supplemented with 0,5% glucose. Bacteria were harvested at 600 nm optical density of 0.25–0.30. 1 ml of encapsulated TIGR4 was centrifuged at 10,000 g for 3 minutes and re-suspended with sterile phosphate buffered saline (PBS) (Lonza, Verviers, Belgium) to a challenge dose of 10^7^ colony forming unit (CFU)/mouse. 1 ml of un-encapsulated TIGR4 was re-suspended in HBMEC/HUVEC cell culture medium to a concentration of approximately 10^7^ CFU/ml.

### Bacteremia derived meningitis model

All experiments involving animals were performed in strict accordance with Dutch legislation on animal experiments (“Wet op de dierproeven”, 1977; modified in 1996 with implementation of the European guidelines 86/609/EEG and “Dierproevenbesluit 1985”) with the prior approval of and in accordance with guidelines of the Institutional Animal Care and Use Committee of the University of Groningen (DEC nr. 6152A). The bacteremia derived meningitis model described by Orihuela et al. [19] was adapted as described before [20].

### Antibodies and lectin

Antibodies and lectin were diluted in sterile PBS with 5% Fetal Calf Serum (FCS) (Biochrom, Berlin, Germany). To detect pneumococci, either an anti-capsule serotype 4 antibody (Statens Serum Institute, Copenhagen, Denmark) or an anti-pneumococcal antiserum [21] (Eurogentec, Maastricht, the Netherlands) 1∶200 diluted were used in combination with an Alexa Fluor 488 goat anti-rabbit antibody (Invitrogen Life Technologies, Carlsbad, United States) 1∶500 diluted. For the detection of endothelial cells, DyLight 594-labeled *Lycopersicon esculentum* Lectin (tomato lectin) (Vector Laboratories, Burlingame, United States) 1∶200 diluted was used [20]. For the detection of nuclei DAPI (Roche, Mannheim, Germany) 1∶5,000 diluted was used. For the detection of human and mouse PAFR, a rabbit anti-PAFR antibody (Cayman Chemicals, Ann Arbor, United States) 1∶50 diluted was used. For the detection of human and mouse pIgR, respectively, a goat anti-human pIgR antibody and a goat anti-mouse pIgR antibody (R & D Systems, Abingdon, United Kingdom) 1∶50 diluted were used. As isotype controls, rabbit IgG (Innovative Research, Plymouth, United States) and goat IgG (Santa Cruz Biotechnology, Dallas, United States) were used at the same dilution as those for specific primary antibodies. For the detection of PAFR and *S. pneumoniae* (both on HBMEC and brain tissue), both primary polyclonal antibodies were labeled using the Zenon Rabbit IgG Labeling Kit (Invitrogen Life Technologies), the anti-PAFR antibody was labeled with Alexa Fluor 350, while the anti-capsule serotype 4 antibody was labeled with Alexa Fluor 488. Subsequently, the labeled antibodies were diluted 1∶50. Isotype controls were labeled with the Zenon Labeling Kit and used at the same dilution as the specific primary antibodies. For the detection of pIgR and *S. pneumoniae,* goat anti-mouse pIgR antibody was combined with an Alexa Fluor 488 donkey anti-goat antibody (Invitrogen Life Technologies), while the anti-capsule serotype 4 antibody and anti-pneumococcal antiserum were labeled with Alexa Fluor 350 with the Zenon Labeling Kit. The goat IgG isotype control was used at the same dilution as used for the anti-pIgR antibody in combination with an Alexa-fluor 488 donkey anti-goat antibody (Invitrogen Life Technologies). To detect pIgR and *S. pneumoniae* on mouse brain tissue by confocal microscopy, the goat anti-mouse pIgR antibody was used in combination with an Alexa Fluor 488 donkey anti-goat antibody (Invitrogen Life Technologies), and the anti-capsule serotype 4 antibody was labeled with Alexa Fluor 488 using the Zenon Labeling Kit. All immunofluorescent steps are summarized in Table S1.

### Immunofluorescent detection

5 µm thin brain/lung sections were fixed with acetone for 10 minutes. After 1 hour incubation with un-encapsulated TIGR4, HBMEC and HUVEC were washed with PBS to remove non-adherent bacteria, fixed with 4% paraformaldehyde (Sigma Aldrich) prior starting the staining procedure. After fixation, cells and tissue sections were incubated with primary antibody for 1 hour at room temperature (RT). After washing with PBS, incubation with secondary antibody for 1 hour at RT followed. To detect nuclei, incubation with DAPI for 10 minutes at RT was performed, slides were then washed with PBS. Citifluor solution (Science Services, Munich, Germany) was added to each tissue section/glass disk. The slides were analyzed with a Leica DM5500B microscope and images were recorded with a Leica DFC 360 FX camera. For confocal imaging A Leica SP2 AOBS microscope was used.

### Image processing

The TIFF images obtained with the 350 nm (blue), 488 nm (green) and 594 nm (red) wavelength filters of the Leica DM5500B fluorescence microscope were merged using the “Color-Merge Channels” ImageJ function [22]. The LEI z-stacks obtained with the confocal microscope Leica SP2 AOBS were merged through Imaris (Bitplane Scientific Software).

### Co-localization analysis

Co-localization of *S. pneumoniae* with pIgR and PAFR was analyzed with ImageJ [22]. The images with the bacterial and pIgR/PAFR signal were opened separately and analyzed with the “Analyze/Co-localization analysis” ImageJ plugin. White pixels were automatically generated on the areas of the bacterial signals co-localizing with the receptor signals. Bacteria that did not co-localize with the pIgR signal remained blue, bacteria not co-localized with the PAFR remained green.

### Bacterial quantification

The surface covered by bacteria was measured using the “Threshold” ImageJ function [22], by determining the area occupied by the 488 nm bacterial signal and the white pixels of the bacteria co-localized with receptors. The percentage of the bacteria co-localized with receptors compared to the total bacterial signal was calculated by dividing the surface area of white bacteria by the total surface area of the green bacteria in each image and multiplication with 100.

### *In vitro* interaction of *S. pneumoniae* with receptors

Lysis buffer was prepared with 50 mM tris-HCl (Promega, Mannheim, GER), 150 mM NaCl, 1% triton X-100, 1% sodium deoxycholate, 0.1% SDS (all from Sigma Aldrich), 1 mM EDTA (Merck Millipore, Billerica, United States), protease inhibitors 1× (Roche). 250 µl of lysis buffer was added to confluent HBMEC or HUVEC grown in T25 flasks (TPP, Trasadingen, Switzerland). Cells were scraped and harvested. After centrifugation at 18,600 g for 15 minutes at 4°C, the cell lysate in the supernatant was harvested. A solution of approximately 10^6^ CFU of un-encapsulated TIGR4 was prepared in PBS. 50 µl of HBMEC or HUVEC lysate was added to 50 µl of the bacterial solution, the mixture was incubated at 4°C with gentle agitation for 1 hour. The mixture was then centrifuged at 18,600 g for 20 minutes at 4°C. The supernatant was removed and the bacterial pellet was washed twice with PBS. The pellet was re-suspended with 100 µl of an anti-pneumococcal antiserum labeled with Alexa Fluor 594 (Zenon Labeling Kit), and incubated at 4°C for 1 hour in the dark. After washing twice with PBS, the bacterial pellet was re-suspended first with an anti-human pIgR antibody solution and incubated at 4°C for 1 hour, and, after washing twice with PBS, secondly re-suspended with an Alexa Fluor Donkey anti-Goat 488 antibody solution and incubated at 4°C for 1 hour in the dark. As a negative control an anti-human α-tubulin antibody was used in combination with an Alexa Fluor 488 Goat anti-Mouse antibody. After the last washing with PBS, finally the bacterial pellet was re-suspended in 100 µl of distilled water. A 5 µl drop was pipetted on a microscope glass slide, covered with a coverslip and analyzed by fluorescence microscopy.

### Pneumococcal adherence to endothelial cells

HBMEC and HUVEC were grown in 12-well plates (TPP) till confluency was reached. When immunofluorescent staining was to be performed after the adherence assay, cells were grown on glass disks placed inside each well. For inhibition assays, cells were grown to confluency at 37°C under 5% CO_2_. To block the receptors, cells were incubated overnight with the respective receptor-specific antibody at a final concentration of 50 µg/ml. As controls, cells were incubated with rabbit IgG or goat IgG also at a final concentration of 50 µg/ml. As a further control, we also used cells that had not been incubated with antibody/IgG. After washing the cells with sterile PBS, 900 µl cell culture medium was added to each well and 100 µl of approximately 10^6^ CFU of un-encapsulated *S. pneumoniae* TIGR4 was added. After 1 hour at 37°C at 5% CO_2_, cells were washed with PBS to remove the non-adherent bacteria and treated with a 50/50 mix of 1% saponin (Merck) and trypsin-EDTA (Gibco) (0.05%–0.02%) and lysed. CFUs were determined by plating serial dilutions of lysed cells on blood agar plates. The ratio of adherent bacteria was calculated by dividing the adherent bacteria by the total amount of bacteria in each well (adherent + non-adherent bacteria).

### Western blotting procedure

Detroit, A549 and HBMEC lysates were prepared as described above (see “In vitro interaction of *S. pneumoniae* with receptors”). Cell lysate proteins were separated by SDS-PAGE using NuPAGE gels (Invitrogen) and blotted (75 min, 100 mA/gel) onto a nitrocellulose membrane (Protran, Schleicher & Schuell, Bath, United Kingdom). Membrane was co-incubated with anti-human pIgR antibody and anti α-tubulin antibody as loading control (see “antibodies and lectin”). PIgR and α-tubulin were then detected using a specific antibody. A mixture of fluorescent IgG secondary antibodies IRDye 800 CW donkey anti-goat (LiCor Biosciences, Bad Homburg, Germany) and 700 CW goat anti-mouse antibody was used for human pIgR and α-tubulin detection, in combination with the Odyssey Infrared Imaging System (LiCor Biosciences). Fluorescence was recorded at 700 and 800 nm.

### Statistical analysis

The independent student t-test of SPSS Statistics 20 (IBM) was used for the statistical analysis of the adherence assay results.

## Results

### PAFR is heterogeneously expressed by the BBB endothelium and *S. pneumoniae* does not co-localize with PAFR

**Figure 1:**
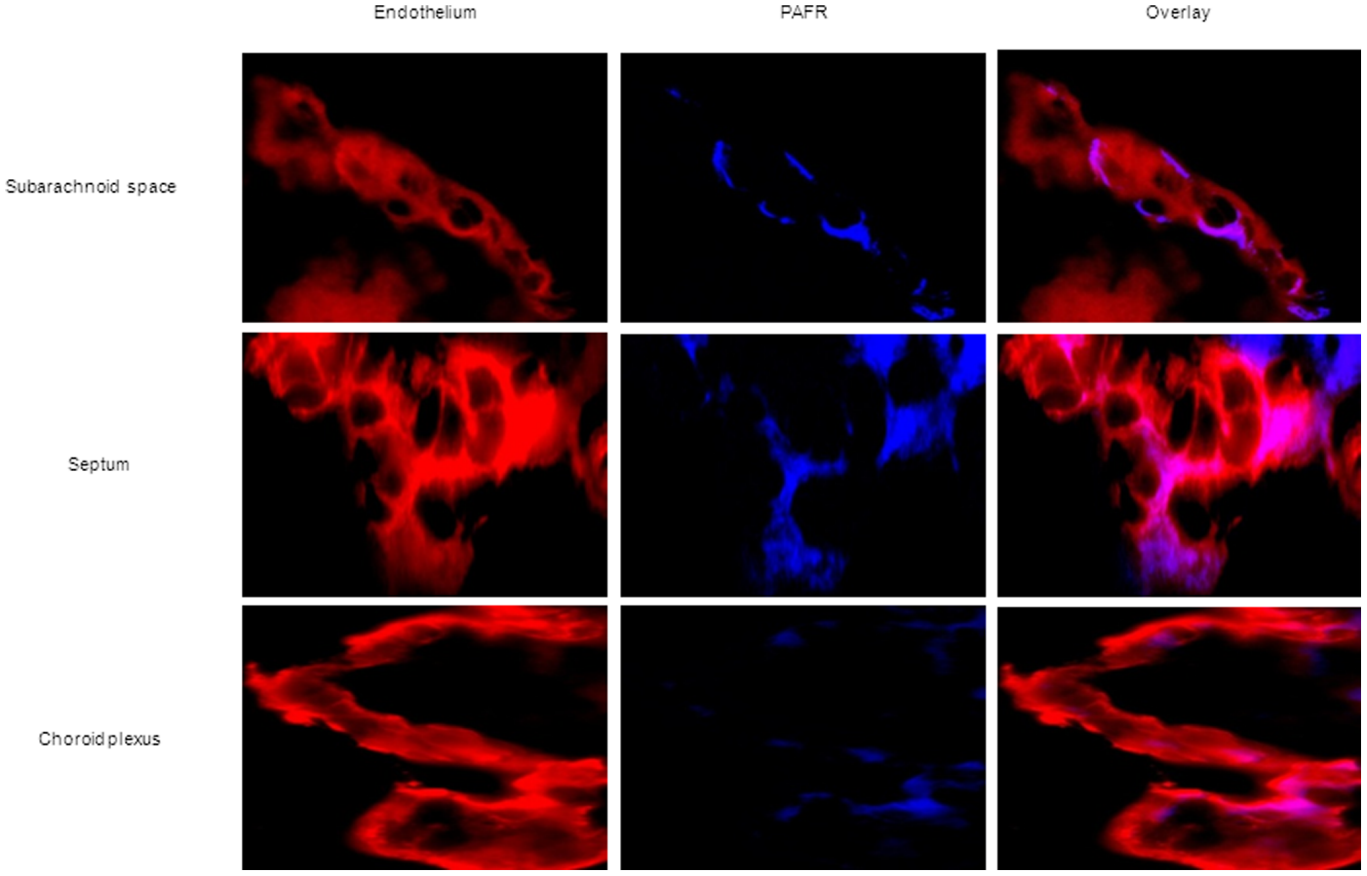
Heterogeneous expression of PAFR by brain endothelium in absence of infection. Immunofluorescent detection of PAFR (blue) and endothelial cells (red) in the different brain compartments of mice in the absence of bacterial infection. Total magnification 630X. Brains from 2 mock infected mice, of each mouse 3 brain sections were used for the immunofluorescent detection. These images are representative of the situation in i) each brain compartment and ii) each mouse that was analyzed.

**Figure 2:**
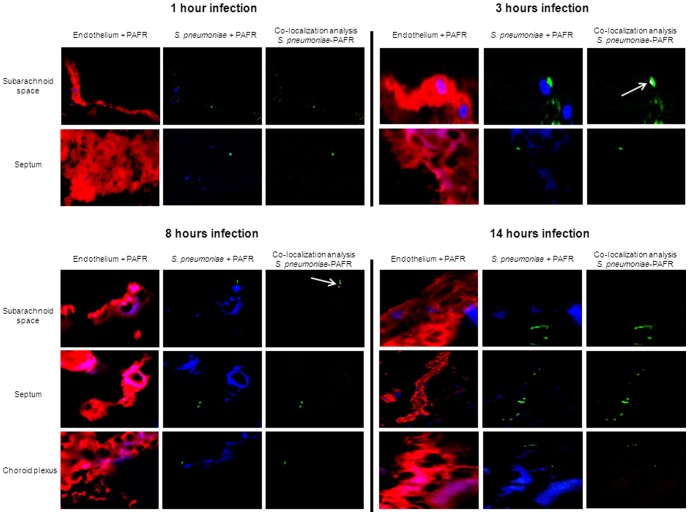
*S. pneumoniae* does not co-localize with PAFR on brain endothelium during the course of infection. Brain slides of mice intravenously challenged with *S. pneumoniae* were stained for vasculature endothelium (red), pneumococci (green), and PAFR (blue). Total magnification 630X. For each time point, the panel “Co-localization analysis *S. pneumoniae*-PAFR” shows bacteria co-localizing with PAFR in white as determined with ImageJ, and bacteria not co-localizing with PAFR in green; white arrows indicate the only pneumococci co-localized with PAFR. For each time point of infection, brains from 3 mice were analyzed, and of each mouse 3 brain sections were used for the immunofluorescent detection. The images are representative of the situation in each brain compartment during the entire time course of infection and in each mouse that was analyzed.

In agreement with previous studies [5], [7], treatment of HBMEC with our anti-PAFR antibody significantly reduced pneumococcal adherence compared to controls (Figure S1A). Immunofluorescent detection of PAFR in HBMEC showed heterogeneous expression by certain clusters of cells (Figure S1B) and analysis using imageJ showed that most pneumococci adherent to HBMEC did not co-localize with PAFR (Figure S1C). In brain tissue of mock-infected mice PAFR was detected mainly on the endothelium although expression was not homogeneous in the various brain compartments (Figure 1). Throughout the time course of infection, most bacteria did not co-localize with PAFR in any of the brain compartments (Figure 2). Semi-quantification of co-localization with ImageJ indicated that <5% of pneumococci co-localized with PAFR at all time points of infection in all brain compartments (Figure 2).

### *S. pneumoniae* co-localizes with pIgR in HBMEC and blockade of pIgR reduces pneumococcal adherence

**Figure 3:**
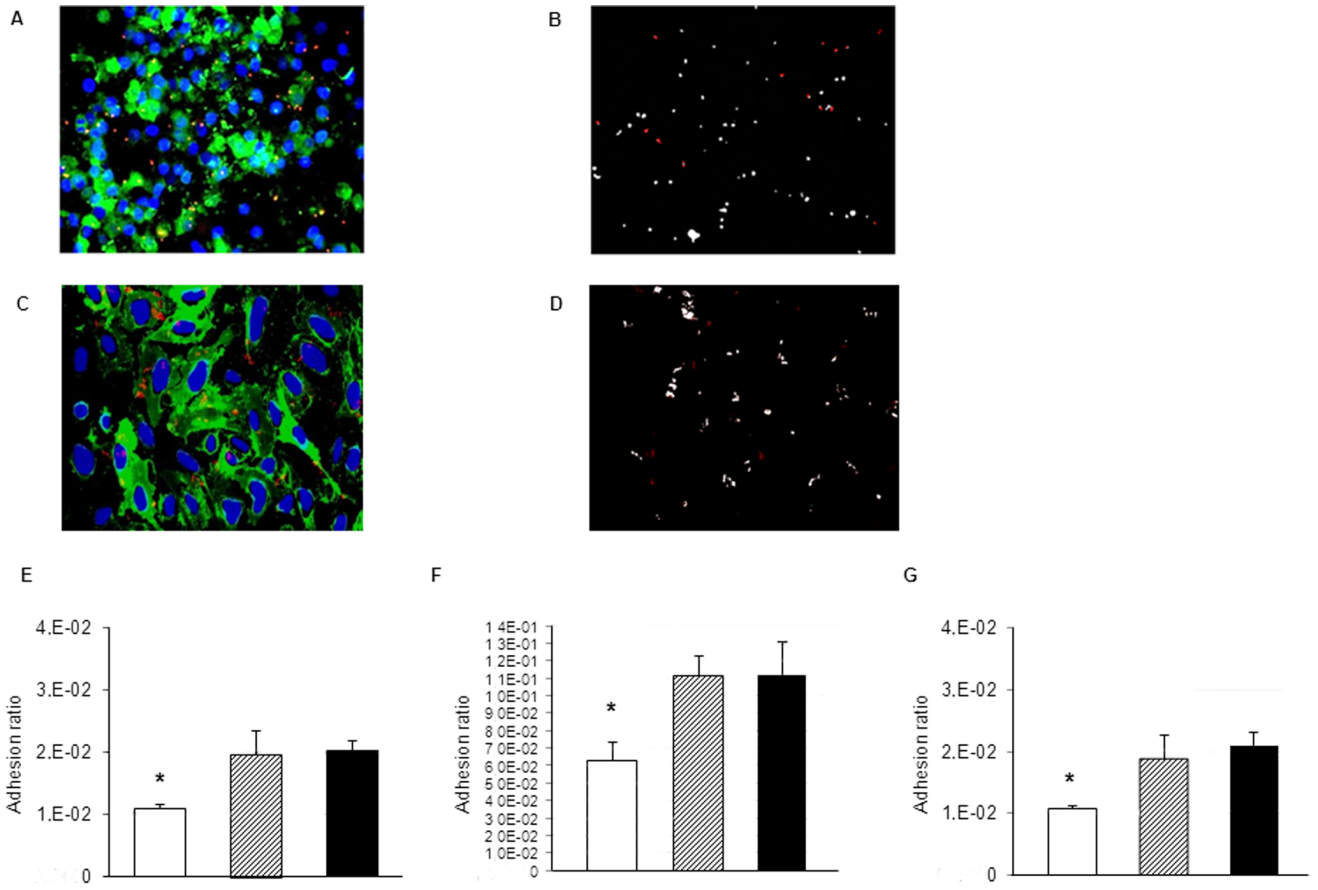
PIgR is involved in *S. pneumoniae* adhesion to human endothelial cells and pneumococci co-localize with pIgR. A. Immunofluorescent detection of pIgR (green), adherent *S. pneumoniae* (red) and cellular nuclei (blue) in HBMEC cells after 1 hour incubation with pneumococci. After 1 hour incubation with pneumococci, HBMEC cells were washed with PBS to remove the non-adherent bacteria and fixed. Total magnification 400X. B. Co-localization between pneumococci and pIgR detected in panel A. White pixels represent the areas of bacterial signal co-localized with pIgR, while red pixels indicate the area of bacterial signal not co-localized with pIgR. C. Immunofluorescent detection of pIgR (green), adherent *S. pneumoniae* (red) and cellular nuclei (blue) in HUVEC cells after 1 hour incubation with pneumococci. After 1 hour incubation with pneumococci, HUVEC cells were washed with PBS to remove the non-adherent bacteria, subsequently immune fluorescent staining was performed. Total magnification 400X. D. Co-localization between pneumococci and pIgR detected in panel C. White pixels represent the areas of bacterial signal co-localized with pIgR, while red pixels indicate the area of bacterial signal not co-localized with pIgR. E-G. Blocking of pIgR (white column) in Detroit cells (E), HBMEC cells (F) and HUVEC cells (G) leads to a significant reduction of pneumococcal adhesion in comparison with Detroit/HBMEC/HUVEC treated with isotype control IgG (hatched column) or with Detroit/HBMEC/HUVEC cells incubated without a blocking antibody (black column). * P value <0.05.

The anti-human pIgR antibody was used for immunofluorescent detection of pIgR in Detroit and A549 cells, respectively known to be positive and negative for pIgR expression [9], [23], and as expected, Detroit expressed pIgR while A549 did not express the receptor (Figure S2). Immunofluorescent analysis showed that pIgR was present on HBMEC (Figure 3A), although endothelial KC cells were reported to not express pIgR [9]. Western blot analysis with the identical anti-human pIgR antibody used for the immunofluorescent analysis detected pIgR in Detroit cells and a band of the same molecular weight in HBMEC. As expected no pIgR expression was observed in A549 and Beas 2b cells [9], [23] (Figure S3), confirming that the immunofluorescent analysis indeed detected pIgR on HBMEC. Most pneumococci adherent to HBMEC co-localized with pIgR as determined by ImageJ (Figure 3B). Similarly, Human Umbilical Vein Endothelial Cells (HUVEC) also expressed pIgR (Figures 3C and Figure S2) and pneumococci adherent to HUVEC also mostly co-localized with pIgR (Figure 3D). Blocking of pIgR using the same antibody significantly reduced adhesion of *S. pneumoniae* to Detroit cells, as previously reported (Figure 3E) [9], and to HBMEC and HUVEC (Figures 3F and 3G) compared to controls.

### *S. pneumoniae* co-localizes with pIgR expressed on the brain vascular endothelium

**Figure 4:**
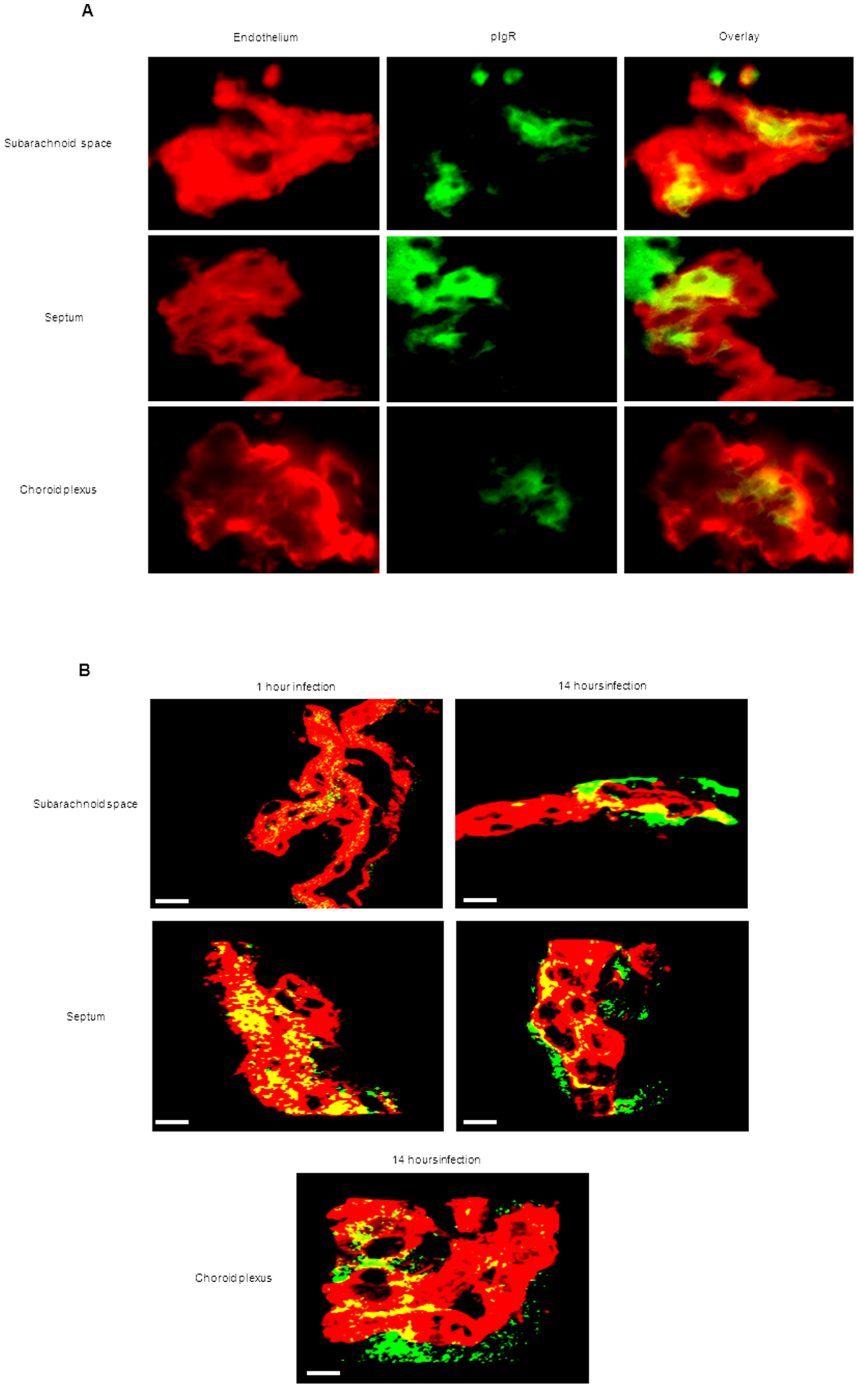
Heterogeneous expression of pIgR by brain endothelium in mock-infected mice. A. Immunofluorescent detection of pIgR (green) and endothelial cells (red) in the brain in absence of bacterial infection shows heterogeneous expression of pIgR on brain endothelium *in vivo*. Total magnification 630X. B. Confocal microscopy images visualizing pIgR (green) on the brain vascular endothelium (red). Overlaps in the pIgR signal and endothelial cell staining resulted in a yellow color, suggestive of pIgR expression by endothelial cells. The white scale bar in each image represents 20 µm. Brains analyzed were from 2 mock treated mice, of each mouse 3 brain sections were used for immunofluorescent detection. These images are representative of the situation in i) each brain compartment and ii) each mouse that was analyzed.

To assess whether the anti-mouse pIgR antibody could be used for immunofluorescent detection of pIgR in mouse tissue, we applied it to lung sections and showed pIgR expression, as was previously reported [9], [24] (Figure S4). PIgR was indeed detected in the healthy mouse brain and associated with endothelial cells (Figure 4A). Analysis of brain sections of mock treated mice, 1 and 14 hours after bacterial challenge using a three-dimensional reconstruction with the computer program Imaris after confocal microscopy confirmed that pIgR expression is indeed associated with endothelial cells (Figure 4B).

**Figure 5:**
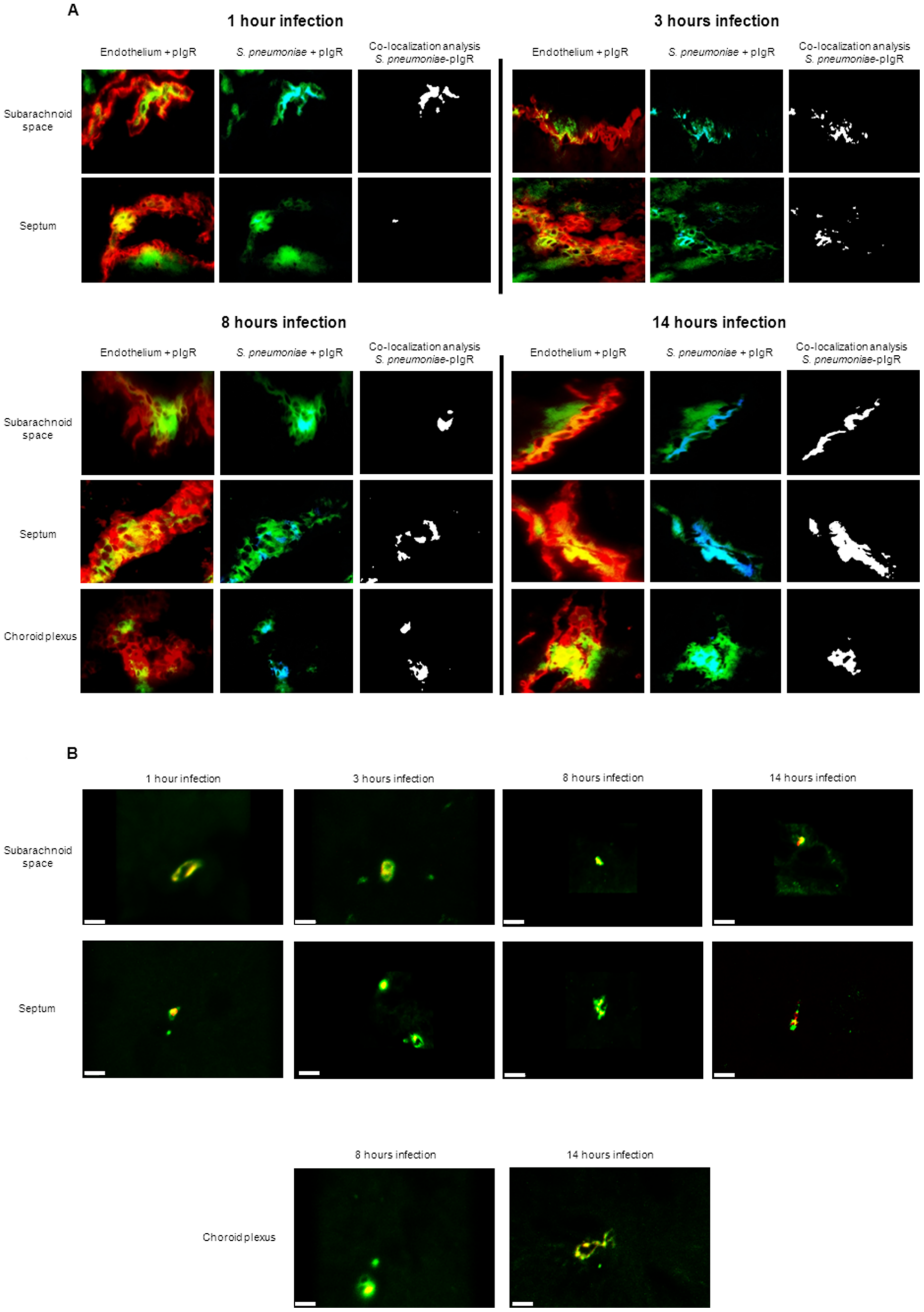
*S. pneumoniae* co-localizes with pIgR on brain endothelium during the entire course of infection. A. Brain slides of mice challenged with *S. pneumoniae* were stained for vasculature endothelium using tomato lectin (red), pneumococci (blue), and pIgR (green). Total magnification 630X. For each time point of infection, brains from 3 mice were analyzed, and of each mouse 3 brain sections were used for the immunofluorescent detection. The images are representative of the situation in each brain compartment during the entire time course of infection and in each mouse that was analyzed. For each time point, the panel “Co-localization analysis *S. pneumoniae*-pIgR” shows bacteria co-localizing with pIgR in white, and bacteria not co-localizing with pIgR in blue. B. Confocal visualization of *S. pneumoniae* (red) and pIgR (green) in the different brain compartments during the whole time course of pneumococcal infection detected by confocal microscopy. The white scale bar in each image represents 5 µm. The yellow color of the bacteria is the result of co-localization of the red-colored *S. pneumoniae* and green-colored pIgR. For each time point of infection, brains from 3 mice were analyzed, and of each mouse 3 brain sections were used for the confocal detection. The images are representative of the situation in each brain compartment during the entire time course of infection and in each mouse that was analyzed.

To investigate whether pneumococci co-localized with pIgR in the brain of infected mice, it was necessary to label the anti-capsule antibody with Alexa Fluor 350. When *S. pneumoniae* was detected with Alexa Fluor 350 (Figure 5A) the coccoid shape and chain structure typical of pneumococci were not always as well distinguishable as when pneumococci were detected with Alexa Fluor 488 (Figure 2). To confirm that the Alexa Fluor 350 signal indeed represented pneumococci, we first stained *S. pneumoniae* in brain tissue with the anti-capsule serotype 4 antibody labeled with Alexa Fluor 350, followed by the same antibody labeled with Alexa Fluor 488. This showed that the bacteria signal detected with Alexa Fluor 350 always co-localized with the signal obtained with Alexa Fluor 488 (data not shown). Notably, in all brain compartments at all time points after infection, more than 95% of pneumococci co-localized with pIgR expressed on the vascular endothelium (Figure 5A). Confocal microscopy confirmed that *S. pneumoniae* indeed co-localized with pIgR (Figure 5B and Data S1).

### *S. pneumoniae* binds to pIgR expressed by human endothelial cells

**Figure 6:**
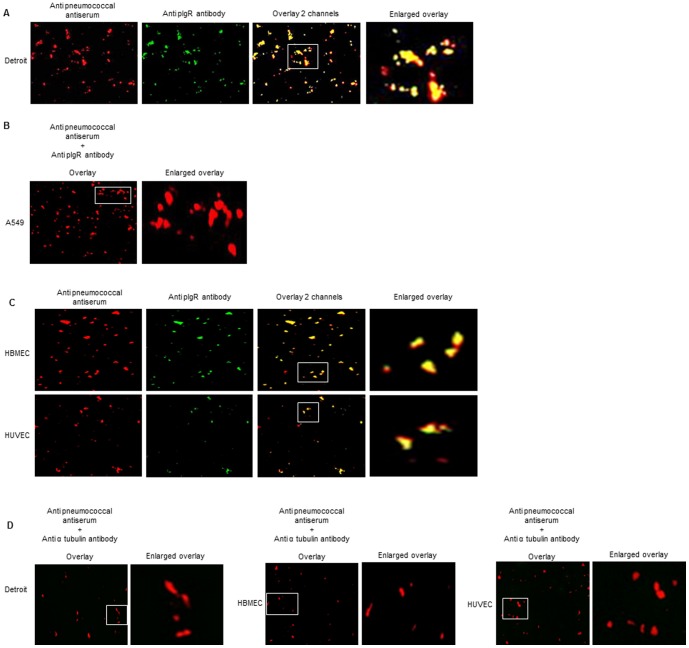
*S. pneumoniae* binds to pIgR ***in vitro***. A. Immunofluorescent detection of *S. pneumoniae* (red) and pIgR (green) after pneumococci were incubated with Detroit. Total magnification 400X. The overlay indicates pIgR signal on the pneumococci. B. Immunofluorescent detection of *S. pneumoniae* (red) and pIgR (green) after pneumococci were incubated with A549 lysate. Total magnification 400X. C. Immunofluorescent detection of *S. pneumoniae* (red) and pIgR (green) after pneumococci were incubated with HBMEC and HUVEC lysates. Total magnification 400X. The overlay indicates pIgR signal on the pneumococci. D. Immunofluorescent detection of *S. pneumoniae* (red) and α tubulin (green) after pneumococci were incubated with Detroit, HBMEC or HUVEC cell lysates. Total magnification 400X.

To assess whether pneumococci could physically interact with pIgR expressed by endothelial cells we set up a ligand-receptor interaction assay (see Material and methods). Since binding of pneumococci to pIgR expressed by Detroit cells was previously described [9], we first tested our method using Detroit cell lysate. After incubation of pneumococci with Detroit cell lysate, pIgR was indeed detected on the bacteria (Figure 6A). As negative control, bacteria were incubated with A549 lysate and probed with anti-human pIgR antibody. As expected by incubating the bacteria with the lysate of a pIgR-negative expressing cells, pIgR was not detected on pneumococci (Figure 6B). *S. pneumoniae* cells incubated with either HBMEC or HUVEC lysates were stained with anti-pneumococcal antiserum and the anti-human pIgR antibody, which showed that in both cases pIgR was present on most bacteria (Figure 6C). As additional control, after incubation with Detroit and endothelial cell lysates the bacteria were probed with an anti-human tubulin antibody. Tubulin was not detected on the pneumococci, indicating that the interaction was specific for pIgR and that endothelial or epithelial proteins did not become associated with the bacteria through non-specific interactions through precipitation/centrifugation (Figure 6D).

## Discussion

The aim of this research was to clarify whether pneumococci adhering to the brain endothelium co-localize with either PAFR or pIgR during the events preceding meningitis. In our present study, co-localization of pneumococci with PAFR was not detectable, neither *in vitro* nor *in vivo*, which leads us to conclude that *S. pneumoniae* is unlikely to bind to PAFR on the BBB. This is in contrast with a study that reported considerable co-localization of PAFR and pneumococci in rat brain endothelial cells *in vitro* [6]. It is conceivable that rBCEC6 express PAFR at higher levels than HBMEC, which would increase the chance of co-localization. However, our finding that the pattern of PAFR expression by HBMEC was similar to that observed in the brains of infected and uninfected mice indicates to us that the PAFR expression observed in HBMEC most likely represents the physiological situation in mice and man. Another difference is that whereas we used anti-capsule antibodies to detect the bacteria, Radin *et al.* used an anti-phosphorylcholine (ChoP) antibody [6], which could in principle also detect ChoP not associated with bacteria. The amino acid sequence of rat PAFR has 79% identity with human PAFR [25] and 91% identity with mouse PAFR [26]. These differences in the PAFR amino acid sequences may also account for the apparent co-localization of pneumococci to PAFR on the rat endothelium and the absence of co-localization with human or murine PAFR.

Although PAFR has been implicated in the adhesion of several pathogens to human cells there is a scarcity of data that demonstrates direct interaction between bacteria and PAFR [8]. Recently, it was shown that *Neisseria meningitidis* is capable of binding to PAFR on human airway cells through the ChoP. As in our study, PAFR was also heterogeneously expressed in bronchial epithelial cells, however 100% of *N. meningitidis* co-localized with PAFR [27]. Furthermore, immune precipitation and ELISA confirmed that *N. meningitidis* binds to PAFR [27]. As we do not observe any co-localization between PAFR and pneumococci in HBMEC and in the brain of mice, and co-localization is a prerequisite for a direct interaction, we consider it highly unlikely that pneumococci bind to PAFR. Thus, it seems that PAFR is a direct receptor for meningococci and an indirect receptor for pneumococci. The inflammation induced by the presence of pneumococci leads to the release of cytokines by the endothelium, including inflammation mediators such as PAF, the ligand of PAFR. The PAFR signaling cascade leads to pro-inflammatory events and the activation of brain endothelial cells might facilitate transmigration of *S. pneumoniae* over cell layers, which would explain the PAFR involvement in IPD [5], [6], [7].

PIgR is a well-known receptor for *S. pneumoniae* in epithelial cells [9], [23], [28], [29], [30]. It has been implicated in the translocation of pneumococci over the epithelium through an intracellular pathway known as transcytosis [9]. Absence of pIgR was reported in human brain endothelial cell line KC [9] and in HUVEC [23], which led to the suggestion that pIgR could exclusively be an epithelial receptor for pneumococci. We detected pIgR in Detroit and not in A549 cells, as reported before [9], and also in HBMEC and HUVEC. The discrepancy concerning brain endothelial cells might be due to the use of different cell lines and different anti-human pIgR antibodies. We used HBMEC while Zhang *et al* used KC cell line, although both are immortalized human brain endothelial cell lines. No data on the absence of pIgR in HUVEC nor information on the provenance of the HUVEC was provided in the manuscript by Agarwal et al [23], whereas we used primary HUVEC isolated in house from different donors and clearly detected a pIgR signal by immunofluorescence and Western Blot analysis. For pIgR detection, Zhang *et al.* prepared a rabbit antiserum against human pIgR and a sheep antiserum against mouse pIgR [9]. The R&D Systems antibodies used in our experiments detect the whole receptor, which has a molecular size of 100–120 kDa, which corresponds to the molecular size of the band detected in our Western blot analysis (Figure S3). To assess the specificity of our anti-human pIgR antibody, we tested the antibody by immunofluorescent staining using Detroit and A549 cells respectively known as positive and negative pIgR-expressing cells [9]. As expected from what was previously reported by Zhang *et al*, Detroit cells showed a relatively high expression of pIgR, while the receptor was not found in A549 cells (Figure S2). The anti-human pIgR antibody was also tested by Western blot analysis, and a pIgR specific band was present in Detroit cell lysate, while A549 did not show any receptor expression (Figure S3). Furthermore, we also included Beas2b cells as additional negative control (Figure S3). Based on the immunofluorescent and Western blot results using control cells such as Detroit, A549 and Beas2b, we concluded that we indeed detected expression of pIgR in HBMEC and HUVEC. In addition, we also demonstrated that pIgR present in human endothelial cell lysates binds to the bacteria, implicating that pIgR may also be involved in bacterial transcytosis of endothelial cells and thus contribute to the development of meningitis.

Several studies show that PspC is a natural ligand for pIgR and is necessary and sufficient for pneumococcal adherence to epithelial cells [28], [29]. Subsequent *in vitro* studies reported that the interaction with PspC was specific for human pIgR [30], [31]. In these studies the interaction between pIgR and PspC was investigated using purified PspC, while we used intact bacteria. The latter might be more relevant and keep the protein in a natural conformation as PspC is normally non-covalently attached to the cell wall through its choline binding motif. Additionally, either isolated soluble component derived from murine pIgR, or transiently transfected cells were used [30], [31], while we specifically detected membrane bound mouse pIgR in endothelial in mouse brain slides and human endothelial cells. Our finding that *S. pneumoniae* co-localized with mouse pIgR is based on the unambiguous analysis of our *in vivo* immunofluorescence and confocal data. Furthermore, the study by Zhang *et al*. [9] clearly showed that absence of pIgR *in vivo* leads to less lung invasion and sepsis, indicating that also in the mouse, interaction between *S. pneumoniae* and pIgR is part of pathogenesis. Further support for a role of (mouse) pIgR comes from studies that show that pneumococci lacking PspC are less adherent to rat BMEC than wild-type, and PspC was shown to be involved in the transition from the lungs to the blood and from the blood into the cerebrospinal fluid (CSF) [9], [19], [32]. This indicates that interaction of PspC to pIgR might be important for the development of meningitis. Alternatively, the interaction between *S. pneumoniae* and endothelial pIgR is mediated through other bacterial proteins.

After intranasal challenge, mice lacking pIgR showed less nasal colonization and decreased levels of bacteremia compared to wild-type mice [9] but, unfortunately, no data was provided on the presence of the bacteria in the brain and or CSF. To definitely assess whether the absence of pIgR significantly reduces bacterial translocation into the brain *in vivo*, intravenous administration of pneumococci in pIgR^−/−^ and WT mice should be performed.

In conclusion, PAFR is unlikely to physically interact with the bacteria *in vivo*. On the other hand, we have shown that pIgR is expressed by brain endothelial cells and may act as a novel receptor for *S. pneumoniae* adhesion to the BBB endothelium. The results presented in this study provide a better understanding of the events preceding pneumococcal meningitis and, in particular, of *S. pneumoniae* receptor-mediated adhesion to the brain microvascular endothelium.

## Supporting Information

Figure S1 PAFR is indirectly involved in *S. pneumoniae* adhesion to human endothelial cells. A. Blocking of PAFR (white column) in HBMEC cells leads to a reduction of pneumococcal adhesion in comparison with HBMEC treated with isotype control (hatched column) and with HBMEC treated without blocking antibody (black column). * P value <0.05. B. Immunofluorescent staining of PAFR (red), adherent *S. pneumoniae* (green) and cellular nuclei (blue) in HBMEC. After 1 hour incubation with pneumococci, HBMEC cells were washed with PBS in order to remove the non-adherent bacteria, after which immune fluorecent staining was performed. Total magnification 400X. C. Co-localization of pneumococci and PAFR detected in panel B. White pixels represent the areas of bacterial signal co-localized with PAFR while green pixels represent the area of bacterial signal not co-localized with PAFR. White arrows indicate the only pneumococci co-localized with PAFR signal observed over all tissue sections of all mice analyzed.
(TIF)

Figure S2 Immunofluorescent detection of pIgR in Detroit and A549 cells. Immunofluorescent detection of pIgR (green) and cellular nuclei (blue) in Detroit (A) and A549 (B) cells. Total magnification 400X.
(TIF)

Figure S3 PIgR detection by Western blotting in Detroit, A549, Beas 2B, HBMEC and HUVEC cells. Expression of pIgR in Detroit (positive control), A549 and Beas 2b cells (negative controls), HBMEC and HUVEC cells was assessed by Western blot analysis using specific antibodies. Simultaneous incubation with alpha tubulin antibody was used as loading control on the same Western blot. The molecular weights of pIgR and alpha tubulin are about 120 kDa and 50 kDa, respectively.
(TIF)

Figure S4 The anti-PIgR antibody detects the receptor on lung epithelial cells. Immunofluorescent detection of pIgR (green) and nuclei (blue) in lungs of mock-treated and infected mice (1 and 14 hours after infection). Total magnification 630X.
(TIF)

Table S1 Immunofluorescent detection scheme.
(DOCX)

Data S1 Three-dimensional visualization of *S. pneumoniae* (red) and pIgR (green) in the subarachnoid space of infected mice detected by confocal microscopy.
(ZIP)
