# Supplementary figures and images for: Streptococcus pneumoniae Interacts with pIgR Expressed by the Brain Microvascular Endothelium but Does Not Co-Localize with PAF Receptor
Source: PLoS One. 2014 May 19;9(5):e97914. doi: 10.1371/journal.pone.0097914 (PMC4026408; doi:10.1371/journal.pone.0097914)

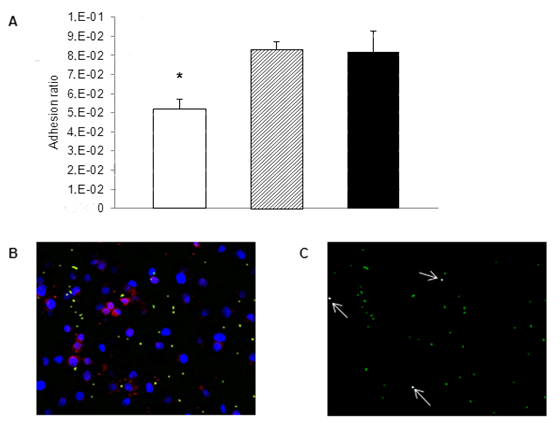

Supplement: Figure S1 — PAFR is indirectly involved in S. pneumoniae adhesion to human endothelial cells. A. Blocking of PAFR (white column) in HBMEC cells leads to a reduction of pneumococcal adhesion in comparison with HBMEC treated with isotype control (hatched column) and with HBMEC treated without blocking antibody (black column). * P value <0.05. B. Immunofluorescent staining of PAFR (red), adherent S. pneumoniae (green) and cellular nuclei (blue) in HBMEC. After 1 hour incubation with pneumococci, HBMEC cells were washed with PBS in order to remove the non-adherent bacteria, after which immune fluorecent staining was performed. Total magnification 400X. C. Co-localization of pneumococci and PAFR detected in panel B. White pixels represent the areas of bacterial signal co-localized with PAFR while green pixels represent the area of bacterial signal not co-localized with PAFR. White arrows indicate the only pneumococci co-localized with PAFR signal observed over all tissue sections of all mice analyzed. (TIF) [file pone.0097914.s001.tif]

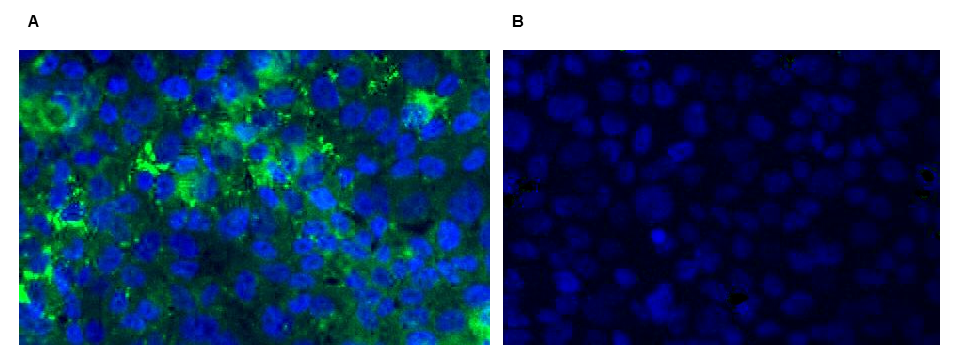

Supplement: Figure S2 — Immunofluorescent detection of pIgR in Detroit and A549 cells. Immunofluorescent detection of pIgR (green) and cellular nuclei (blue) in Detroit (A) and A549 (B) cells. Total magnification 400X. (TIF) [file pone.0097914.s002.tif]

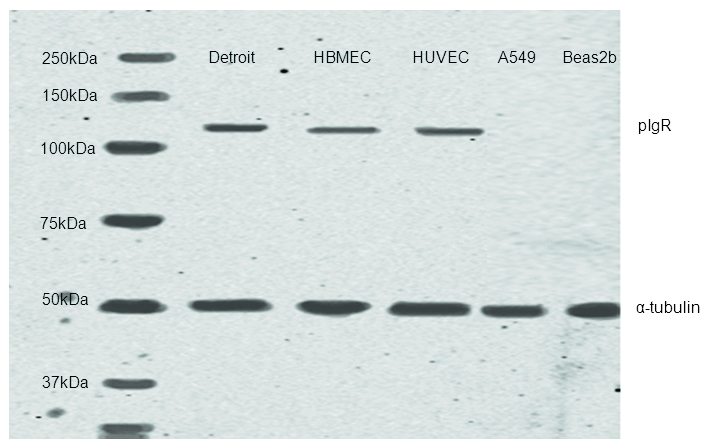

Supplement: Figure S3 — PIgR detection by Western blotting in Detroit, A549, Beas 2B, HBMEC and HUVEC cells. Expression of pIgR in Detroit (positive control), A549 and Beas 2b cells (negative controls), HBMEC and HUVEC cells was assessed by Western blot analysis using specific antibodies. Simultaneous incubation with alpha tubulin antibody was used as loading control on the same Western blot. The molecular weights of pIgR and alpha tubulin are about 120 kDa and 50 kDa, respectively. (TIF) [file pone.0097914.s003.tif]

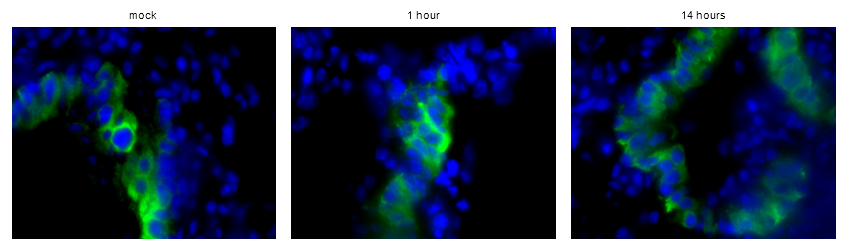

Supplement: Figure S4 — The anti-PIgR antibody detects the receptor on lung epithelial cells. Immunofluorescent detection of pIgR (green) and nuclei (blue) in lungs of mock-treated and infected mice (1 and 14 hours after infection). Total magnification 630X. (TIF) [file pone.0097914.s004.tif]
