# Supplementary material for: Streptococcus pneumoniae Interacts with pIgR Expressed by the Brain Microvascular Endothelium but Does Not Co-Localize with PAF Receptor
Source: PLoS One. 2014 May 19;9(5):e97914. doi: 10.1371/journal.pone.0097914 (PMC4026408; doi:10.1371/journal.pone.0097914)
Supplement: Table S1 — Immunofluorescent detection scheme. (DOCX) [file pone.0097914.s005.docx]

**Table S1 . Immunofluorescent detection scheme**

**Incubation steps**

| **Detection of PAFR and *S. pneumoniae* in brain tissue** | **1^st^**  Anti PAFR antibody labeled with Alexa Fluor 350 (Zenon Kit)  Dilution 1:50 | **2^nd^**  Anti capsule serotype 4 antibody labeled with Alexa Fluor 488 (Zenon Kit)  Dilution 1:500 | **3^rd^**  tomato lectin  Dilution 1:200 | **4^th^**  DAPI  1:5000 |
| --- | --- | --- | --- | --- |
| **Detection of pIgR and *S. pneumoniae* in brain tissue** | **1^st^**  Anti capsule serotype 4 labeled with Alexa Fluor 350 (Zenon Kit)  Dilution 1:50 | **2^nd^**  Anti pIgR antibody  Dilution 1:50 | **3^rd^**  Mixture tomato lectin and Alexa Fluor 488 Donkey anti Goat  Dilution 1:500 | **4^th^**  DAPI  1:5000 |
| **Detection of pIgR in lung tissue** | **1^st^**  Anti pIgR antibody  Dilution 1:50 | **2^nd^**  Alexa Fluor 488 Donkey anti Goat  Dilution 1:500 | **3^rd^**  DAPI  Dilution 1:5000 |  |
| **Detection of PAFR and *S. pneumoniae* in HBMEC and HUVEC** | **1^st^**  Anti PAFR antibody labeled with Alexa Fluor 594 (Zenon Kit)  Dilution 1:50 | **2^nd^**  Anti capsule serotype 4 antibody labeled with Alexa Fluor 488 (Zenon Kit)  Dilution 1:50 | **3^rd^**  DAPI  1:5000 |  |
| **Detection of pIgR and *S. pneumoniae* in HBMEC and HUVEC** | **1^st^**  Mixture of anti pIgR antibody (dilution 1:50) and anti capsule serotype 4 antibody (dilution 1:200) | **2^nd^**  Mixture of Alexa Fluor 488 Donkey anti Goat and Alexa Fluor 594 Goat anti Rabbit  Dilution 1:200 | **3^rd^**  DAPI  Dilution 1:5000 |  |
